# FOSTERING MULTIDISCIPLINARY SOLUTIONS IN AGING: THE RESEARCH CENTERS COLLABORATIVE NETWORK

**DOI:** 10.1093/geroni/igae098.1167

**Published:** 2024-12-31

**Authors:** Stephen Kritchevsky, Odette van der Willik

**Affiliations:** Chair; Co-Chair

## Abstract

The NIA supports the Research Centers Collaborative Network (RCCN) to bridge scientists from the 7 NIA-sponsored center programs: Alzheimer’s Disease Research Centers, Artificial Intelligence and Technology Collaboratories for Aging Research, Centers on the Demography and Economics of Aging, Claude D. Pepper Older Americans Independence Centers, Nathan Shock Centers of Excellence in the Basic Biology of Aging, Resource Centers for Minority Aging Research, and Roybal Centers for Translational Research on Aging. To provide a forum for collaborative exchange, RCCN has convened workshops on crosscutting concerns relevant to multiple NIA center programs ranging from sustaining behavior change in older adults to measuring biologic age. RCCN also awards pilots that address topics spanning the missions of multiple NIA Center Programs. This symposium will present key learnings from the work of five RCCN pilot teams. Dr. King will discuss a novel analysis of proteomic and cellular senescence biomarkers in kidney using a non-human primate model. Dr. Nguyen will discuss the relationship between social disconnection and changes in cognitive health over time among older Black adults. Dr. Quiñones-Cordero will discuss culturally congruent interventions to improve Latino ADRD caregivers’ psychological well-being. Dr. Christensen will discuss the development and implementation of older adult community engagement studios (OA-CES) to promote the inclusivity of older adults in research and to increase the number of research studies on older adults. Dr. Landauer will discuss the development of Age Friendly training webinars and research tools. Dr. Kritchevsky will also summarize lessons learned across the workshops.

